# Supplementary material for: Rare Diseases: Needs and Impact for Patients and Families: A Cross-Sectional Study in the Valencian Region, Spain
Source: Int J Environ Res Public Health. 2022 Aug 19;19(16):10366. doi: 10.3390/ijerph191610366 (PMC9408677; doi:10.3390/ijerph191610366)
Supplement: Supplementary file 1 [file ijerph-19-10366-s001.zip › ijerph-1865368-supplementary.pdf]

INFORMED CONSENT

TITLE OF THE STUDY:
"Analysis, determinants and impact of diagnostic delay in rare diseases"

PRINCIPAL INVESTIGATOR:
Óscar Zurriaga. Department of Universal Health and Public Health. FISABIO-Public Health
Tel: 961925727; email: zurriaga_osc@gva.es

CENTRE: FISABIO

INFORMED CONSENT FORM

I, ...,

(as legal representative of..),

with identification number..

I have read the information sheet given to me.
I have been able to ask questions about the study.
I have received sufficient information about the study.
I understand that my participation is voluntary.
I understand that I can withdraw from the study:

- whenever I want.
- without having to explain.
- without affecting my medical care.

I freely agree to participate in the study.

Date: ....

Signature of the participant or legal representative of the minor (mother, father or legal guardian):

....

In the case of mature minors (12 years or older), is there assent from the minor?

☐ No

☐ Yes

Signature of the mature minor (12 years of age or older):

....

TO SEND

This questionnaire is addressed to people affected by rare diseases.

QUESTIONNAIRE ON DIAGNOSTIC DELAY

**If the person concerned is a minor and/or has a disability or difficulty in completing this questionnaire, the answers will be completed on their behalf. If you have any questions please contact enfermedades_raras1@gva.es or 961926327.*

Who filled in the questionnaire?

- ☐ Person concerned
- ☐ Mother
- ☐ Father
- ☐ Partner
- ☐ Other person (specify:______________________)

SOCIO-DEMOGRAPHIC DATA OF THE PERSON WITH RARE DISEASE (PATIENT)

- ✓ Sex:
  - ☐ Female
  - ☐ Male
  - ☐ Other
- ✓ Nationality: ____________________
- ✓ Date of birth_________________________
- ✓ Place of residence: ______________________
- ✓ Education level (completed):
  - ☐ No formal education
  - ☐ Primary school education
  - ☐ High school education
  - ☐ University studies
  - ☐ Other (please specify): ________________

DETAILS OF THE FAMILY UNIT WHERE THE PATIENT LIVES

Taking into account all the MONTHLY income of the household, please indicate the total amount or choose one of the options below. The household's income during 2018 was:

MONTHLY FAMILY TOTAL:__________________________€

- ☐ No income
- ☐ Less than 400 euros net per month
- ☐ Between 401 and 800 euros net per month
- ☐ Between 801 and 1,200 euros net per month
- ☐ Between 1,201 and 2,000 euros net per month
- ☐ Between 2,001 and 2,800 euros net per month
- ☐ Between 2,801 and 3,600 euros net per month
- ☐ More than 3,600 euros net per month

- ✓ In total, for how long have family unit members worked in the last 12 months?

**Person who has worked** (patient, mother, father, sibling, partner, child etc.)	**No. of hours worked per week**	**No. of months worked**

☐ We have not worked (with remuneration) during the last 12 months

Do you feel that your household is struggling to make ends meet?

☐ Yes ☐ No

Last month, in which of the following employment situations were the persons (*18 years and older)* in the household? *(Specify your relationship to the patient: mother, father, grandmother, grandfather, sibling...)*

How many people live in the household? ☐

Patient is:________________________________
(Me, son/daughter, mother, father, partner, sibling...)
- ☐ Employed (full or part-time)
- ☐ Unemployed. Since (date)___/__/___
- ☐ Student or trainee
- ☐ Retiree, early retiree, pensioner
- ☐ Permanently incapacitated for work / disabled
- ☐ Investor, landlord
- ☐ Domestic housework and/or care of children or other persons (unpaid)
- ☐ Other types of inactive persons

Member 1 is: ______________________________
(Mother, father, partner, sibling, grandparent...)
- ☐ Employed (full or part-time)
- ☐ Unemployed. Since (date)___/__/___
- ☐ Student or trainee
- ☐ Retiree, early retiree, pensioner
- ☐ Permanently incapacitated for work / disabled
- ☐ Investor, landlord
- ☐ Domestic housework and/or care of children or other persons (unpaid)
- ☐ Other types of inactive persons

Member 2 is:______________________________
(Mother, father, partner, sibling, grandparent...)
- ☐ Employed (full or part-time)
- ☐ Unemployed. Since (date)___/__/____
- ☐ Student or trainee
- ☐ Retiree, early retiree, pensioner
- ☐ Permanently incapacitated for work / disabled
- ☐ Investor, landlord
- ☐ Domestic housework and/or care of children or other persons (unpaid)
- ☐ Other types of inactive persons

Member 3 is: ______________________________
(Mother, father, partner, sibling, grandparent...)
- ☐ Employed (full or part-time)
- ☐ Unemployed. Since (date)___/___/____
- ☐ Student or trainee
- ☐ Retiree, early retiree, pensioner
- ☐ Permanently incapacitated for work / disabled
- ☐ Investor, landlord
- ☐ Domestic housework and/or care of children or other persons (unpaid)
- ☐ Other types of inactive persons

Member 4 is: ______________________________
(Mother, father, partner, sibling, grandparent...)
- ☐ Employed (full or part-time)
- ☐ Unemployed. Since (date)___/___/____
- ☐ Student or trainee
- ☐ Retiree, early retiree, pensioner
- ☐ Permanently incapacitated for work / disabled
- ☐ Investor, landlord
- ☐ Domestic housework and/or care of children or other persons (unpaid)
- ☐ Other types of inactive persons

Member 5 is: ______________________________
(Mother, father, partner, sibling, grandparent...)
- ☐ Employed (full or part-time)
- ☐ Unemployed. Since (date)___/___/____
- ☐ Student or trainee
- ☐ Retiree, early retiree, pensioner
- ☐ Permanently incapacitated for work / disabled
- ☐ Investor, landlord
- ☐ Domestic housework and/or care of children or other persons (unpaid)
- ☐ Other types of inactive persons

Member 6 is:______________________________
(Son/daughter, mother, father, partner, sibling...)
- ☐ Employed (full or part-time)
- ☐ Unemployed. Since (date)___/___/____
- ☐ Student or trainee
- ☐ Retiree, early retiree, pensioner
- ☐ Permanently incapacitated for work / disabled
- ☐ Investor, landlord
- ☐ Domestic housework and/or care of children or other persons (unpaid)
- ☐ Other types of inactive persons

Member 7 is:______________________________
(Son/daughter, mother, father, partner, sibling...)
- ☐ Employed (full or part-time)
- ☐ Unemployed. Since (date)___/___/____
- ☐ Student or trainee
- ☐ Retiree, early retiree, pensioner
- ☐ Permanently incapacitated for work / disabled
- ☐ Investor, landlord
- ☐ Domestic housework and/or care of children or other persons (unpaid)
- ☐ Other types of inactive persons

**Please indicate whether the reality of your household matches the resources and situations listed below:**	**Yes, this describes my household situation.**	**No, this doesn't describe my household situation.**	**No, but it is a voluntary decision**
The family is in arrears on rent, mortgage, housing-related bills or instalment purchases.	☐	☐	
The home is kept at a comfortable temperature during the cold months.	☐	☐	
The family can cope with unforeseen expenses.	☐	☐	
The family eats meat, poultry or fish at least every other day.	☐	☐	☐
The family goes on holiday away from home for at least one week a year.	☐	☐	☐
There is a family car.	☐	☐	☐
There is a washing machine.	☐	☐	☐
There is a colour television.	☐	☐	☐
There is a telephone.	☐	☐	☐
The family can pay for other treatments (rehabilitation, speech therapists, physiotherapy...) if needed.	☐	☐	
The family can pay for supportive therapies or psychological care in private consultations if needed.	☐	☐	
The family can pay a caregiver to help them if they need it.	☐	☐	

DATA RELATED TO THE DIAGNOSIS:

✓ Do you have a currently confirmed diagnosis of a Rare Disease?
- ☐ Yes, I have a confirmed diagnosis.
- ☐ I have a diagnosis, but it is not confirmed (under test or pending results).
- ☐ I have no diagnosis.

Specify the **name of the Rare Disease**:

- ✓ Year and month of onset of first symptoms:

- ✓ Year and month of diagnosis:

- ✓ Which professional made the diagnosis of the Rare Disease? (specify: neurology, digestive, internal medicine etc.)
  - ☐ Family doctor (primary care)
  - ☐ Paediatrician at the health centre
  - ☐ Specialist (digestive, paediatrician, neurologist, ophthalmologist, cardiologist, dermatologist etc.) Indicate:____________

- ✓ Treatments **BEFORE** **diagnosis**:
  - ☐ **Drug therapy (**medicine). Indicate the **type** (name) of the medicine and how it was **taken** (at home, at the health centre, in hospital, etc.).
  - ☐ **Other treatments** (e.g. eyeglasses, creams, rehabilitation, physiotherapy, speech therapist etc.). Indicate the **type** (name) and how many **times** a week.
  - ☐ **Alternative therapies** (e.g. homeopathy, acupuncture, healing diets, Reiki etc.). Indicate the **type** (name) and how many **times** a week the therapy was done.

☐ **Medicinal plants and/or herbal products.** Indicate the **type** (name) and how many **times** a week the substance was taken.

✓ Treatment **AFTER diagnosis**:

☐ **Drug therapy (**medicine). Indicate the **type** (name) of the medicine and how it was **taken** (at home, at the health centre, in hospital, etc.).

☐ **Other treatments** (e.g. eyeglasses, creams, rehabilitation, physiotherapy, speech therapist etc.). Indicate the **type** (name) and how many **times** a week.

☐ **Alternative therapies** (e.g. homeopathy, acupuncture, healing diets, Reiki etc.). Indicate the **type** (name) and how many **times** a week the therapy was done.

☐ **Medicinal plants and/or herbal products**. Indicate the **type** (name) and how many **times** a week the substance was taken.

✓ The **current** pharmacological (drug) **treatment** for your Rare Disease is:

- ☐ Chronic (long-term)
- ☐ Temporary (planned end date)
- ☐ Occasional (outbreaks or relapses)
- ☐ I am **not** currently undergoing treatment
- ☐ Other. Indicate:___________________________

Please indicate if you have suffered from any of the following situations:	Due to the delay in diagnosis	As a result of the disease
1. Hospitalisation		
2. Aggravation of the pathology		
3. Travel to other centres and/or specialists		
4. Employment repercussions* (Temporary leave, reduction of working hours, dismissal etc.)		
5. Disruption of personal relationships (partner, family, friends)		
6. Separation, divorce or break-up of the relationship		
7. Mood disturbance		
8. Disruption of daily routine		
9. Poor or reduced social support network		
10. Social rejection or discrimination against you		

*(Non-voluntary)

✓ Appearance of the first symptoms of Rare Disease:

- ☐ Physical effects (cutaneous, ocular, alteration of the senses, injuries etc.)
- ☐ Psychological effects (anxiety, depression, sleep disturbances, etc.)
- ☐ Motor impairment (alteration of gait, movement, speech, language, etc.)
- ☐ Multiple effects
- ☐ Other effects. Indicate:_____________________________

✓ Needs arising from the Rare Disease and which are not available (by the health system) and cannot be paid for:

- ☐ Medical treatment / health care
- ☐ Psychological support
- ☐ Physiotherapeutic support
- ☐ Medicines and other healthcare products
- ☐ Technical aids / orthopaedics
- ☐ Personal support or assistance
- ☐ Adaptation of housing
- ☐ Adaptation of the workplace in the work location
- ☐ Non-clinical co-adjuvant therapies (equine therapy, music therapy, swimming etc.)
- ☐ Access to leisure and free time activities
- ☐ Other (please specify):_______________________

✓ Monthly (approximate) financial costs of the disease: ____________________________€
(including treatment and other therapies and/or products)

- ☐ There is **no** financial cost to me (the treatment is subsidised).

✓ Your financial expenses in relation to your Rare Disease are distributed in:

- ☐ Drug therapy
- ☐ Therapy (physiotherapy, speech therapy, rehabilitation etc.)
- ☐ Psychological support and/or care
- ☐ Alternative medicine (acupuncture, homeopathy etc.)
- ☐ Support person (domestic and/or personal help)
- ☐ Specialised care centres

✓ In comparison to prior to the diagnosis, I would say that the family's financial situation: (please tick only one answer)

- ☐ It has become much worse.
- ☐ It has worsened somewhat.
- ☐ It has remained the same.
- ☐ It has improved a little.
- ☐ It has improved a lot.

If you have any comments and/or other contributions, please write them below:

Thank you for your participation and your time!

Date:______________________
